# Microcavity-integrated graphene waveguide: a reconfigurable electro-optical attenuator and switch

**DOI:** 10.1038/s41598-018-30396-8

**Published:** 2018-08-20

**Authors:** Guorong Sui, Jun Wu, Yuehua Zhang, Chenhui Yin, Xiumin Gao

**Affiliations:** 1 Shanghai Key Laboratory of Modern Optical System, Engineering Research Center of Optical Instrument and System (Ministry of Education), University of Shanghai for Science and Technology, Shanghai, 200093 China; 2 School of Electrical Engineering and Automation, Hainan University, Hainan, 570228 China

## Abstract

Graphene has been widely utilized in optoelectronic applications due to its high carrier mobility, and extremely fast optical response. Microcavity-integrated graphene waveguide structure is one basic module of integrated photonic devices which can greatly improve the light-matter interaction strength. The enhanced optical absorption in the undoped graphene layer results from the light trapping and the corresponding long light-graphene interaction length. Tuning the Fermi energy level of the graphene layer enables the electro-optical modulation. We report the realization of reconfigurable electro-optical attenuator and switch with unity-order modulation depth in light reflection and transmission at near-infrared frequency. The transformation from a lossy absorber to a quasi-perfect transparent condition of the monolayer graphene by tuning the Fermi level leads to the unity-order tunability of the electro-optical attenuator and switch. We investigate theoretically and numerically the absorption properties of the designed microcavity-integrated graphene with respect to different graphene Fermi levels. Electro-optical attenuator with attenuating coefficient from 10% to 98.29% is fulfilled. On-off electro-optical switching with a switching contrast larger than 21 dB is demonstrated. Our approach provides the possibilities of graphene photonics applied in communications, and sensing.

## Introduction

The intriguing optoelectronic and photonic properties of graphene has led to an increasing research interest in utilizing graphene for electro-optical applications. Generally, for monolayer graphene, the interaction strength with light is rather weak due to the inherent single atom thickness (the corresponding optical absorption coefficient is 2.3% which is independent of wavelength^1^). The weak optical absorption is detrimental to the realization of high-performance graphene based optoelectronic device^2–6^, where absorption up to 100% is desired^4,7,8^. Specifically, to develop optical devices such as photodetectors and optical antennas, high optical absorption in graphene is required to generate sufficient large photocurrents. Boosting the light-matter interaction strength in graphene is one important issue which needs to be addressed to take further advantage of graphene in optoelectronic devices. Microcavity structure has been demonstrated to possess excellent performance in confining light in the defect layer and hence greatly boosts light-matter interaction strength^9^. The light-matter interacting enhancement only occurs at the designed resonant wavelength, which makes it promising for the wavelength division multiplexing system^10^. For graphene, one important property is its optical absorption can be easily manipulated^11–14^. The conductivity of the graphene layer is highly sensitive to the Fermi level. By shifting the electronic Fermi level, one can controllably change graphene’s optical transitions to achieve unexpected tunable waveguide devices. The linear dispersion of the graphene Dirac fermions provides an ultra-wideband tunability by applying electrostatic field, magnetic field, or chemical doping.

In this work, we investigate the tunability of reflection\transmission properties of a microcavity-integrated graphene waveguide structure by comprehensively studying the interaction between the monolayer graphene and the microcavity under different graphene Fermi levels in the near-infrared region to achieve reconfigurable electro-optical attenuator and switch. The nearly unity absorption of the incident light in the graphene layer is achieved by critically coupling the light into a guided resonant mode of the Fabry–Pérot cavity. The microcavity also allows us to suppress the absorption to be almost zero through actively adjusting the graphene Fermi level. Taking advantage of the operation of the critical coupling condition and resonant tunneling effect of the microcavity, we demonstrate a unity-order variation range in both reflection and transmission. The unity-order modulation depths in the microcavity-integrated graphene waveguide provide guidelines for further development of novel graphene-based passive components.

## Results

### Microcavity-integrated graphene structure

The absorption coefficient of a monolayer graphene depends on its unique band structure and many other parameters including scattering rate, operating temperature, electron velocity, as well as electric and magnetic field bias^15–22^. For electromagnetic wave propagating normal to graphene, the wave absorption coefficient depends strongly on the graphene conductivity, the value of which can be tuned efficiently with the implementation of different Fermi levels. Note that, graphene Fermi level (*E*_*F*_) of 1.2 eV and 1 eV have been experimentally achieved through electrostatic doping^23^ and chemical doping^24^. The refractive index of the surrounding media, between which graphene is located, also affect the graphene’s absorption characteristics. Normally, the absorption coefficient for the geometry with graphene acting as a conducting interface sandwiched between two dielectric media with refractive indices of *n*_1_ and *n*_2_, respectively, at different operating frequencies can be calculated utilizing the boundary conditions from Maxwell’s equations as^25^:

1 $$A(\omega )=\frac{4\cdot real(\sigma (\omega ))\cdot {Z}_{0}/{n}_{1}(\omega )}{{(1+{n}_{2}(\omega )/{n}_{1}(\omega )+real(\sigma (\omega ))\cdot {Z}_{0}/{n}_{1}(\omega ))}^{2}+(imag(\sigma (\omega ))\cdot {Z}_{0}/{n}_{1}(\omega ){)}^{2}},$$

where *ω* is the angular frequency, *Z*_0_ = 120*π* Ohm is the free-space impedance and *n*_1_(*ω*) and *n*_2_(*ω*) are refractive indices of the surrounded dielectrics. The optical conductivity *σ*(*ω*) of graphene represents the electron transition and determines the electromagnetic wave absorption coefficient *A*(*ω*).

**Figure 1:**
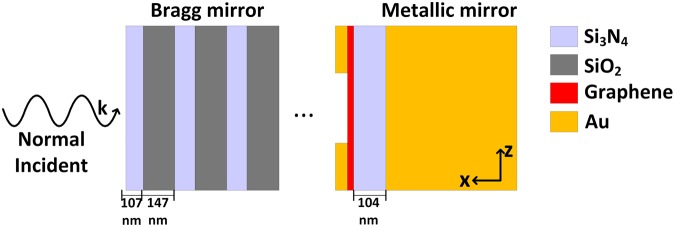
Schematic of the two-dimensional microcavity-integrated graphene waveguide incorporating a graphene layer inside the cavity (the cavity is flanked by a top lossless Bragg mirror and a bottom metallic mirror). The normal incident light is trapped in the defect region and passes multiple times through the graphene layer to greatly increase the light-graphene interaction strength. The source and drain Au electrodes are deposited to modify the graphene Fermi level value.

Microcavity offers a convenient method of actuation for electro-optical modulators: tunable optical characters of the integrated absorber layer can lead to the manipulation of the optical features of the incident light. One more attractive characteristic of the microcavity structure is its resonant response occurs only at wavelengths that are an integer fraction of the effective optical path length of the cavity. Great light confinement and enhancement in cavity provide strong light-matter interaction for optical routing with the need of relative lower input incident energy. Figure 1 shows the two-dimensional schematic illustrations and basic geometric parameters of the microcavity integrated-graphene waveguide structure. The microcavity-integrated graphene waveguide with a distributed top Bragg mirror (consisting of quarter-wavelength thick layers of alternating dielectric materials with varying refractive indices), a thick bottom metallic mirror, and a defect layer forms a high-finesse Fabry–Pérot cavity. Bragg mirror is an ideal choice for microcavity optoelectronic devices because the reflection can be well controlled from zero to unity. The features of the tunable absorption of the graphene layer will add more flexibility for the graphene-based monolithic integrated device. To achieve high light absorption, we place the graphene sheet inside a symmetric Fabry–Pérot cavity, with a perfect back metallic mirror and a partially transmitting front Bragg mirror. We consider a defect layer between the Bragg mirror and the metallic mirror to produce a single resonant wavelength at 850 nm. The Bragg mirror is composed of alternating silicon nitride (Si_3_N_4_) and silica (SiO_2_) layers with thickness of 107 nm and 147 nm, and the bottom metallic mirror is set to be a 200 nm gold film (to make sure the reflection reaches to unity). The monolayer graphene is integrated into the defect region acting as an active electrochromic material. Note that, the selected materials of the Bragg mirror and the defect layer are non-absorbing at the resonant wavelength to provide high optical performance of the microcavity. The defect Si_3_N_4_ layer guarantees the maximum trapped electric field amplitude occurs right at the graphene sheet position. The thickness of the Si_3_N_4_ defect layer is set to be 104 nm.

**Figure 2:**
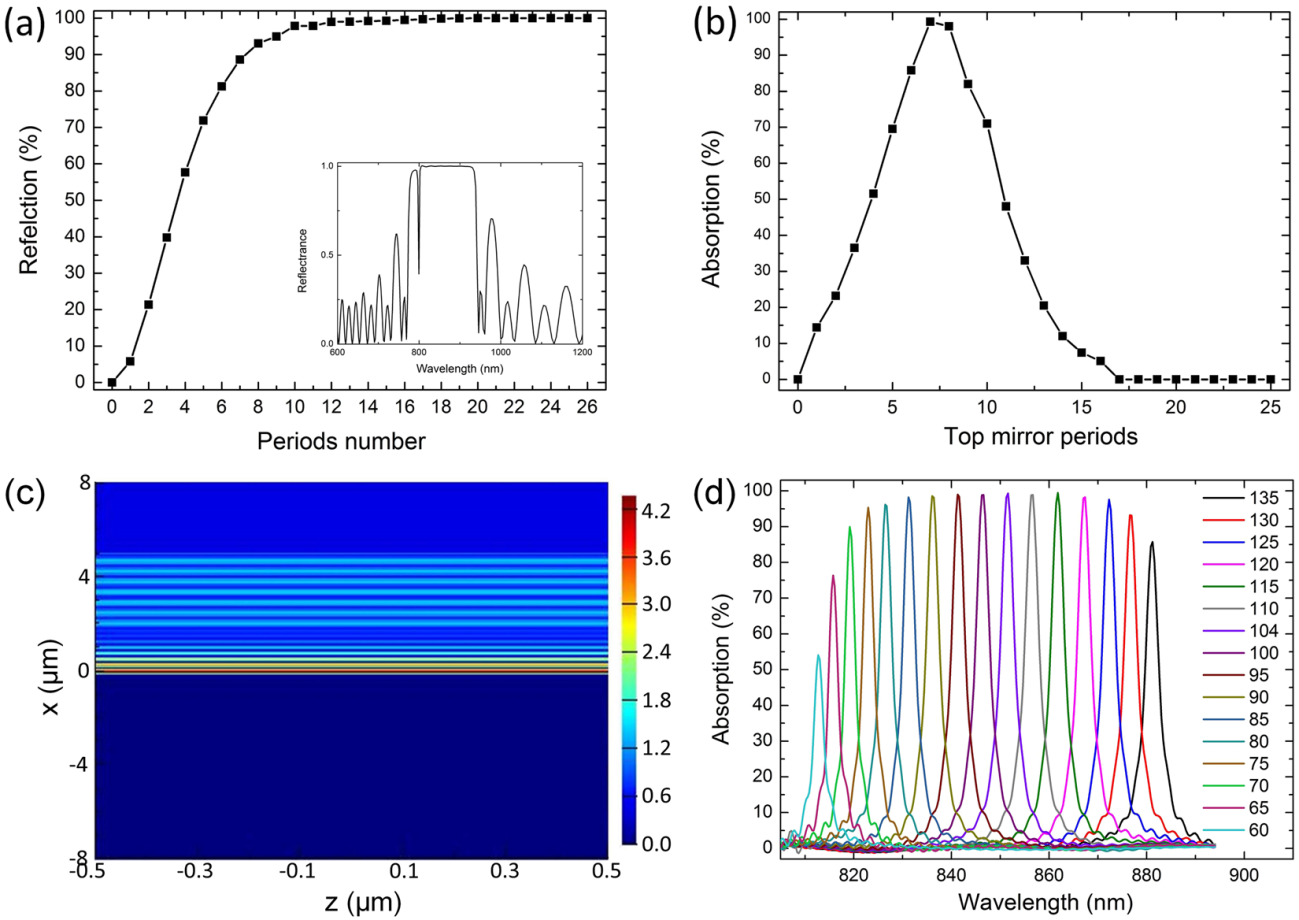
Optical properties of the Bragg mirror with various periods. (**a**) Reflections of the Bragg mirror with various period numbers. (**b**) The absorption coefficient of the microcavity-integrated graphene with different periods of the top Bragg mirror. (**c**) Electric field amplitude inside the cavity at resonance. (**d**) Defect layer thickness dependent absorption of the microcavity-integrated graphene waveguide. The defect layer thickness is changed from 60 nm to 135 nm.

The microcavity is considered to be critically coupled when the leakage rate of a guided resonant mode out of the Fabry–Pérot cavity is equal to the absorption rate in the graphene layer and all the incident light is absorbed correspondingly. We use finite different time domain (FDTD) method to simulate the optical behaviors of the Bragg mirror and the microcavity-integrated graphene waveguide to achieve critical coupling. Three-dimensional FDTD simulations are performed with periodic boundary conditions along the y and z directions and prefect matching layer (PML) condition along the x direction. During the simulation, the dynamical graphene optical conductivity is calculated using the random-phase approximation method as

2 $$\begin{array}{c}\sigma (\omega )=\frac{2i{e}^{2}{k}_{B}T}{\pi {\hslash }^{2}(\omega +i{\tau }^{-1})}\,\mathrm{ln}[2\,\cos (\frac{{E}_{F}}{2{k}_{B}T})]\\ \,\,\,+\frac{{e}^{2}}{4\hslash }\{0.5+\frac{1}{\pi }\arctan (\frac{\hslash \omega -2{E}_{F}}{2{k}_{B}T})-\frac{i}{2\pi }\,\mathrm{ln}[\frac{{(\hslash \omega +2{E}_{F})}^{2}}{{(\hslash \omega -2{E}_{F})}^{2}+{(2{k}_{B}T)}^{2}}]\}\end{array}$$

where *k*_*B*_ is the Boltzmann constant, *T* is the temperature, *ω* is the frequency, *τ* is the carrier relaxation time from the impurities, and *E*_*F*_ is the Fermi level. The in-plane graphene permittivity is characterized as $\varepsilon =2.5+i\sigma (\omega )/({\varepsilon }_{0}\omega t)$. The other dielectric materials are modeled as lossless dielectric materials with refractive indices reported in^26–29^. The reflections of the Bragg mirror with different period numbers are depicted in Fig. 2(a). The reflection increases with increasing period number, and reaches to unity when the period number reaches to 12. The refractive index contrast between SiO_2_/Si_3_N_4_ offers the Bragg mirror with reflection up to unity in a broad spectral range around 850 nm as the inset shows in Fig. 2(a).

Then we optimize the waveguide geometrical parameters for critical coupling through the transfer matrix method. The thickness of the bottom gold film mirror is firstly fixed to be 200 nm to ensure a perfect suppression on the incident light transmission. Next, the period number of the top Bragg mirror is optimized to cancel the reflection to obtain a unity-order absorption. Figure 2(b) shows the absorption coefficient of the microcavity-integrated graphene with respect to different top Bragg mirror periods. The absorption increases with increasing the reflection (period number) of the top Bragg mirror and reaches a maximum of 100% when the period number comes up to seven and drops to zero as the reflection of the Bragg mirror approaches to unity. For larger reflection of the top mirror, i.e., the period number of the top Bragg mirror is more than seven, the cavity is too lossy and the field enhancement in the cavity reduces to be extremely small. When the reflection of the top Bragg mirror increases to 100%, all of the incident light is reflected on the incident surface and cannot enter into the cavity, thus the absorption is decreased down to zero. Since the graphene layer is in-plane isotropic, the optical response here is independent of light polarization at normal incidence. The unity-order absorption indicates that a 43-fold increment of light intensity is achieved inside the defect cavity region. An equivalent interpretation of the significant absorption in graphene can be stated as: the incident photons bounce between the bottom and top mirrors and pass multiple times through the graphene. Figure 2(c) shows the resonant electric field distribution at wavelength of 850 nm. Graphene is placed at x = 0. We calculated the defect layer thickness dependent absorption of the microcavity-integrated graphene waveguide (Fig. 1 in manuscript), As shown in Figure R2. The cavity resonance red-shift with increasing Si_3_N_4_ defect layer thickness. The varied cavity resonance and total absorption are due to the different interference strength of the counter-propagating incident and reflected waves at different defect layer thickness conditions. According to our calculation, the Q-factor (Q = λ/Δλ) of the resonant mode in the microcavity structure can reach as high as 328, which can be further enhanced by further increasing the defect layer thickness or selecting other defect material with higher refractive index (the trend of the Q-factor follows $2\pi nL/\lambda$, where *n* is the refractive index of the defect layer, *L* is the corresponding thickness, and *λ* is the free space wavelength). Owing to the broadband absorption of graphene, the concept of critical coupling at 850 nm also extends to other wavelengths by tuning the thicknesses of the quarter-wavelength alternating dielectric layer of the top Bragg mirror and the defect layer.

### Reconfigurable electro-optical attenuator and switch on reflection

Continuous variable optical attenuators are key components of fiber optic communication networks and have been commonly used either to test power level margins or to control the intensity of one or more light wavelengths. One challenge within the current optical systems is to fabricate attenuator that is flexible, lightweight, reliable, and has a minimum amount of moving parts. Optical switching technology is intensively driven by the need to provide flexibility in optical network connectivity. In telecommunication, an optical switch is a device that enables signals in optical fibers or integrated optical circuits to be selectively switched from one circuit to another. We expand the concept of electro-optical attenuating and switching of graphene absorption by coupling to a high Q-factor microcavity. In the microcavity-integrated graphene waveguide, we assume no plasmonic response in the graphene layer. The light trapping and enhancement in the cavity region of the microcavity allows the strong interaction between the monolayer graphene and the incident light. The concept of the unity-order absorption model reminds us the tuning of absorption of the microcavtiy to achieve optical modulator by adjusting the absorption of the graphene layer. The critical coupling effect is robust, and the frequency where total absorption occurs can be tuned by adjusting either the waveguide geometric parameters or the material properties. The method through tuning the conductivity of the graphene to obtain absorption tunability by employing different graphene Fermi levels is much easier since graphene can be tuned from highly lossy to transparent or vice visa by tuning its Fermi level in visible and near-infrared region.

In order to gradually tune the reflection to the greatest extend from zero to unity-order, the bottom mirror should be as close to perfect reflector as possible, since any transmission through it represents energy leakage, and this energy portion cannot be absorbed. With undoped monolayer graphene integrated into the cavity, full absorption of the normally incident light can always be obtained at resonant wavelength condition. Under this total absorption condition, when a proper doping condition applied on the graphene, the lossy graphene layer can be tuned to be transparent wherein the light can directly reach the bottom perfect mirror that reflects all of the incident light. Once we achieved de-critical coupling situation by tuning the Fermi level, we can obtain the reflection of the microcavity-integrated graphene waveguide larger than zero and even up to unity-order.

**Figure 3:**
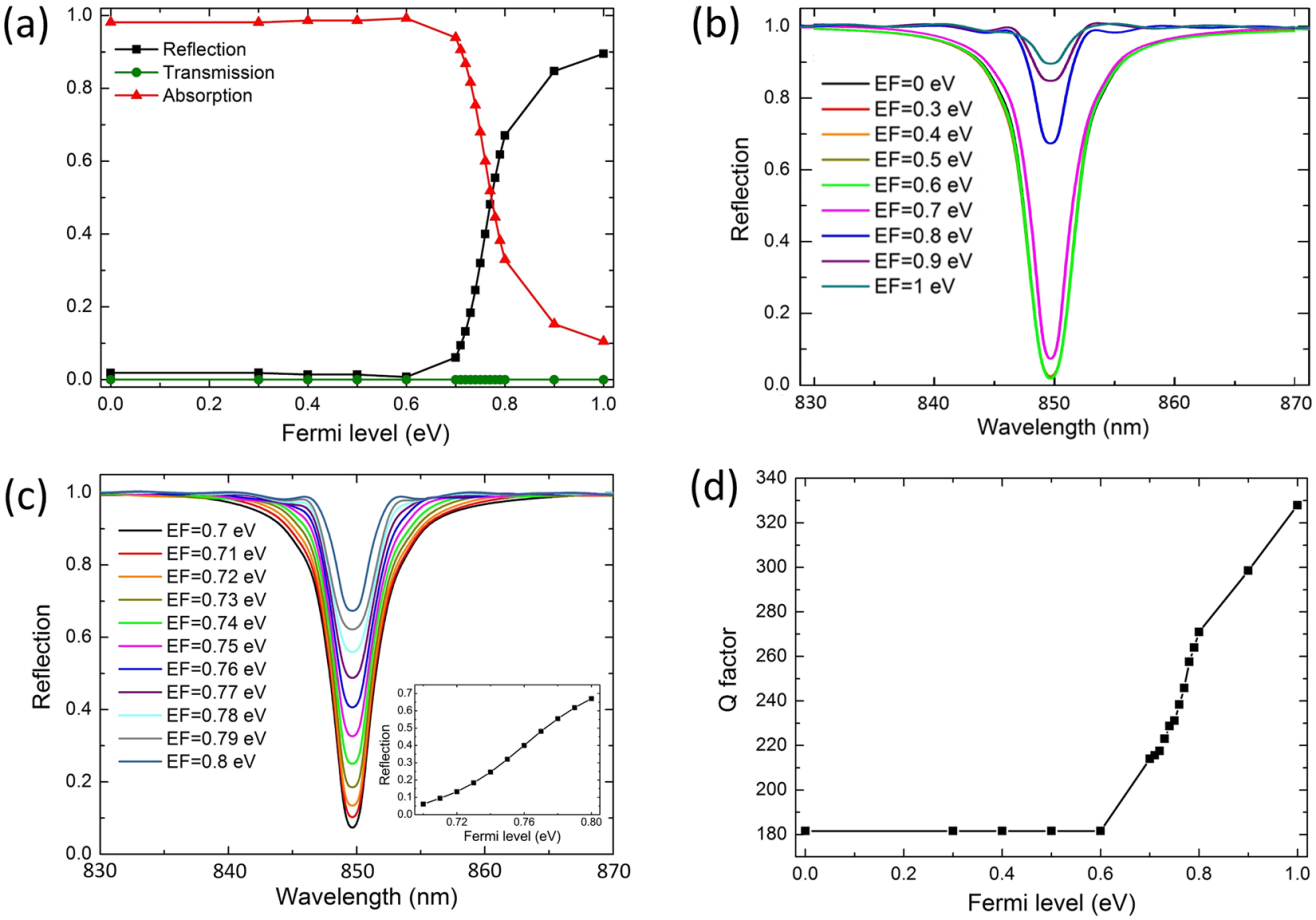
(**a**) Normal incidence reflection, transmission and absorption of the microcavity-integrated graphene for different Fermi levels. (**b**) Reflection spectrum for graphene Fermi level changing from 0 eV to 1 eV with interval of 0.1 eV. (**c**) Reflection spectrum for graphene Fermi level changing from 0.7 eV to 0.8 eV with interval of 0.01 eV. The insertion shows the reflection values at different Fermi levels. (**d**) Q-factor of the microcavity-integrated graphene at different Fermi levels.

Figure 3(a) displays the reflection (R), transmission (T) and absorption (A) spectra of the 850 nm photons passing through the microcavity with different graphene Fermi levels. Under *E*_*F*_ = 0, graphene can absorb all of the incident electromagnetic waves corresponding to A ≈ 1, R ≈ 0 and T = 0 corresponding to a unity-order light confinement factor. When the graphene layer is highly doped (*E*_*F*_ = 1 eV), it becomes nearly lossless, and the peak reflection reaches 90%. As shown in Fig. 3(a), the attenuating coefficient of the microcavity integrated-graphene waveguide can be tuned from 98.29% to 10% continuously by properly tuning the graphene Fermi level from 0.6 eV to 1 eV, which means that the reflection can be tuned from 1.71% to 90% gradually. The increment in reflection when moving from undoped to highly doped graphene condition is only due to the change of absorption coefficient of the monolayer graphene. Therefore, the microcavity undergoes unity-order modulation on the attenuating coefficient upon graphene doping. Figure 3(b,c) illustrate the reflection spectrum with different Fermi level conditions in detail. Figure 3(c) shows clearly the gradual modulation on the reflection by tuning the graphene Fermi level from 0.7 eV to 0.8 eV. In fact, the real part of the graphene conductivity determines the modulation depth whereas the imaginary part is responsible for the resonance wavelength shift. The imaginary part of the graphene conductivity corresponds to the real part of the permittivity. As can be seen from Fig. 3 (b,c), because the graphene thickness is modeled to be 0.5 nm, with Fermi level tuned from 0 eV to 1 eV, the permittivity of the graphene does not change too much to effect the guided resonant mode distribution in the cavity, where the resonant wavelength exists nearly non-shift with increasing doping level. Figure 3(d) shows the Q-factor of the microcavity-integrated graphene structure as a function of graphene Fermi level. Higher Fermi level leads to larger Q-factor, which can also be observed from Fig. 3(b,c) (higher Fermi level corresponds to narrower bandwidth). Note that, to tune the graphene Fermi level, source and drain Au electrodes as shown in Fig. 1 are needed which can be deposited by laser lithography, E-beam evaporation, and lift-off. The substrate acts as a gate electrode. The graphene Fermi level can be modified by applying different gate voltages.

The obtained switching effect is as following: for the microcavity-integrated graphene electro-optical switch, a connection is in the on state if the graphene is highly doped with essentially 10% loss in optical energy and 90% reflection. A connection is in the off state if essentially zero reflection emerges. The reflection coefficient is tunable from −21.02 dB at *E*_*F*_ = 1 eV to −0.48 dB at EF = 0 eV, resulting in an electro-optical switching contrast of 20.54 dB.

### Reconfigurable electro-optical attenuator and switch on transmission

**Figure 4:**
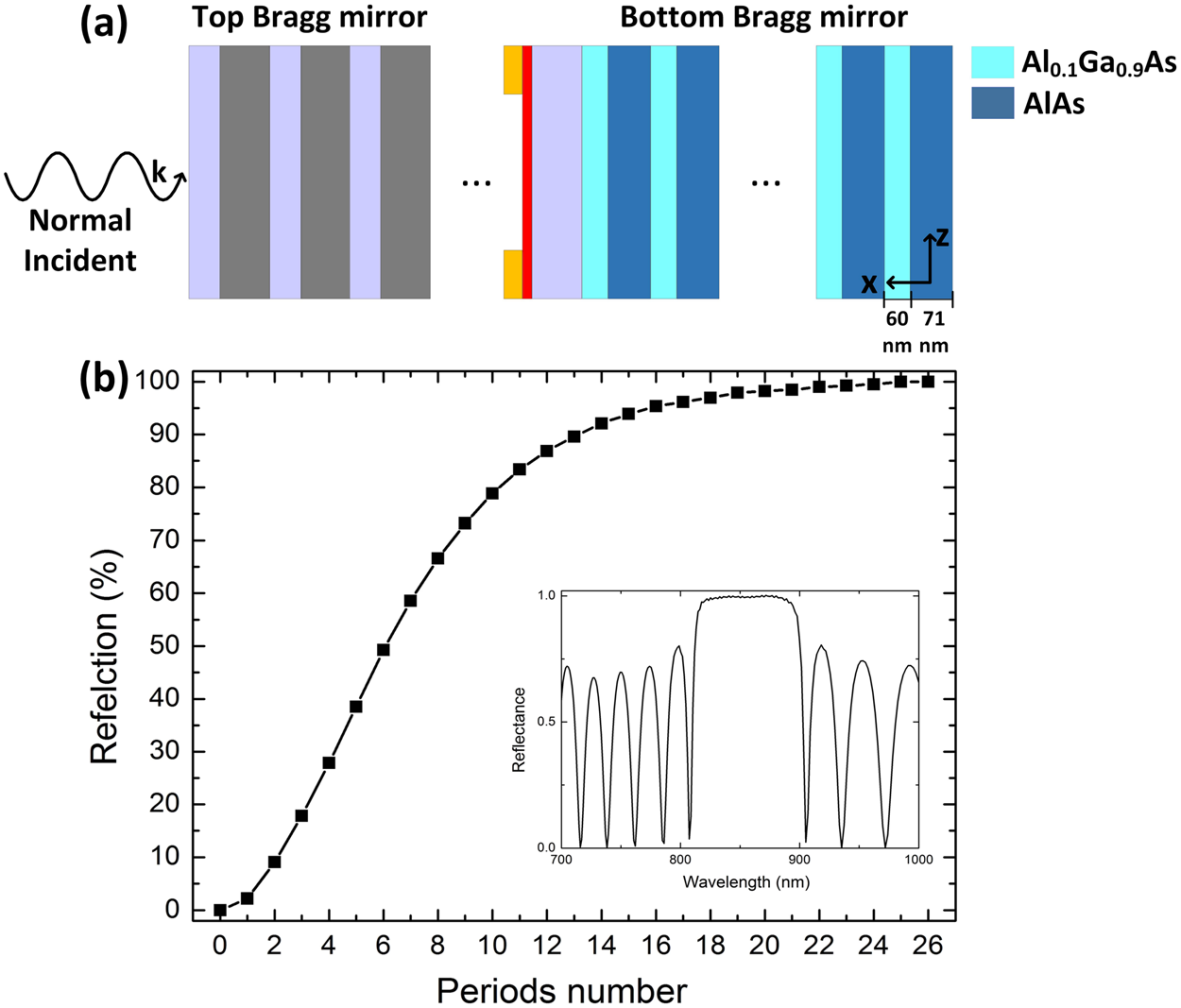
Schematic structure and Optical guiding of two-dimensional microcavity-integrated graphene waveguide. (**a**) Schematic of two-dimensional microcavity-integrated graphene waveguide incorporating a graphene layer inside the cavity flanked by two lossless Bragg mirrors. (**b**) Reflection of the bottom Bragg mirror with various period numbers.

The concept of the tunable reflection of the microcavity also applies to other types of resonators in which the incident field undergoes a significant enhancement in the cavity decorated with graphene. With two non-absorbing, nearly perfect reflecting Bragg mirrors as shown in Fig. 4(a), the transmission of the microcavity without graphene can approach to be as large as unity due to the tunneling effect, based on which we desire high modulation depth of an electro-optical attenuator and switch. The top Bragg mirror is composed of alternating silica (SiO_2_) and silicon nitride (Si_3_N_4_) layers with same geometrical parameters shown in Fig. 1, and the bottom Bragg mirror is composed of alternating aluminum gallium arsenide (Al_0.1_Ga_0.9_As) and aluminum arsenide (AlAs) layers with thickness of 60 nm and 71 nm, respectively. The monolayer graphene is integrated into the defect Si_3_N_4_ region (same geometrical parameter shown in Fig. 1) acting as an active electrochromic material. Figure 4(b) indicates that when the period number of the bottom Bragg mirror comes up to 25, its reflection reaches to unity. The refractive index contrast between AlAs/Al_0.1_Ga_0.9_As offers the back Bragg mirror with reflection up to unity in a broad spectral range around 850 nm as well.

**Figure 5:**
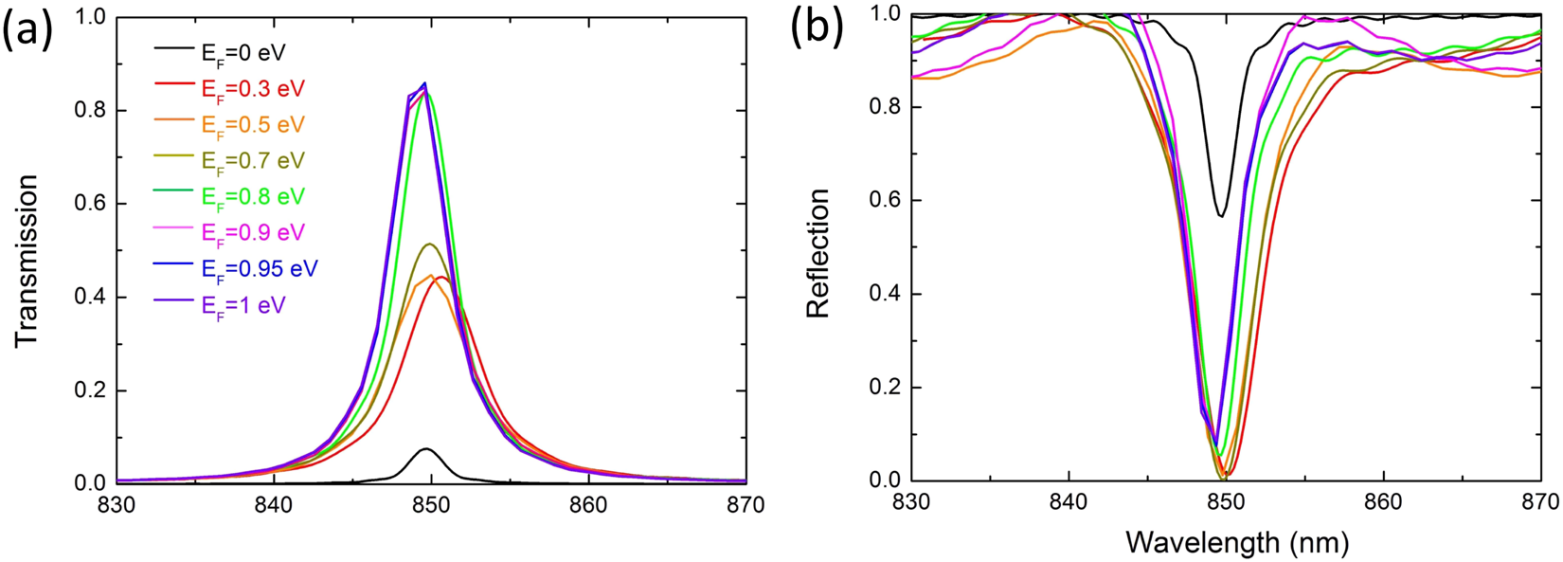
Transmission and reflection spectra of the Fabry–Pérot resonator at different Fermi levels. (**a**) Transmission and (**b**) reflection with different graphene Fermi levels for the resonant Fabry–Pérot cavity with normal incidence.

Simulation results indicate that the implementation of the tunneling effect can be satisfied by setting the period numbers of the top and bottom Bragg mirrors of 8.5 and 19, respectively. Under complete transmission condition, the incident field has to be strongly amplified in the cavity. At resonance, the incident light traps inside the cavity and passes through the graphene layer multi-times similar to the above. Without graphene, the typical full width at half-maximum of the transmission spectrum is 3.3 nm and the Q-factor is 257.6. The transmission and reflection spectra of this Fabry–Pérot resonator at different Fermi levels are shown in Fig. 5(a,b). The transmission can be tuned between two extreme values of 0.08 and 0.85 by varying the graphene Fermi level, resulting in a switching contrast of 21.42 dB. Therefore, the Fabry–Pérot resonator undergoes unity-order modulation of the transmission upon graphene doping. Notice the blue-shift of transmission with increasing the Fermi level on the graphene essentially mimics the effect of the imaginary part of the graphene conductivity with doping.

## Discussion

In conclusion, we theoretically demonstrate the integration of a monolayer graphene layer into a microcavity to produce unity-order tunability in reflection, transmission and absorption of the normally incident light in the near-infrared domain by means of tunable critical coupling with the guided resonant mode. The modulation depths in light reflection and transmission in the two different microcavity-integrated graphene structures exceeding 90% and 75%, respectively, indicating small insertion losses. This attenuator/switch is configured to be compact, and lightweight while proving wavelength agility and tunability at chip-level, and can be used on platforms having limited size, weight, and power budgets. Due to the broadband tunable absorption of graphene, the proposed concept can extend to visible, mid-infrared and far-infrared domains by appropriately selecting the alternating dielectric layer thickness of the Bragg mirror and the thickness of the defect layer. Compared with the conventional electro-optical attenuators and switches, the microcavity-integrated graphene optical attenuator and switch provide a new path to develop graphene-based nano-scaled photonics with fast optical modulation speed, low electric power consumption, and easy integration with large-scale photonic array without complex packaging techniques, pointing to the applications in communications, sensing, security, and spectroscopy.
